# Resilience of ant communities (Hymenoptera: formicidae) to moderate urban disturbance in a mediterranean peri-urban landscape

**DOI:** 10.1007/s00114-026-02152-w

**Published:** 2026-09-10

**Authors:** Athanasia Ntouzou, Jakovos Demetriou, Eleftheria Kyriazidi, Anastasia Misseyanni, Christos Georgiadis

**Affiliations:** 1 Section of Zoology – Marine Biology, Department of Biology, Faculty of Science, National and Kapodistrian University of Athens, Athens, Greece; 2 Section of Ecology and Systematics, Department of Biology, Faculty of Science, National and Kapodistrian University of Athens, Athens, Greece; 3 Department of Natural Sciences, School of Science and Technology, The American College of Greece, Athens, Greece; 4 Zoology Museum, Department of Biology, Faculty of Science, National and Kapodistrian University of Athens, Athens, Greece

**Keywords:** Ants, Anthropogenic disturbance, Community composition, Intermediate disturbance hypothesis, Urbanization, Peri-urban forest

## Abstract

Human activities alter the biotic and abiotic characteristics of the environment, affecting species richness, population abundances and community composition. Nevertheless, these activities intensify as we move from forested areas to the center of urban cities, with peri-urban areas presenting intermediate trends. In this study, we investigate the role of anthropogenic disturbance to ant community composition and diversity in the peri-urban forest of Agia Paraskevi (Attica), Greece. Sampling was conducted during a full year (June 2022 - May 2023) using pitfall traps, both in natural areas and in areas under human influence. Pitfalls from four months (i.e. January, April, July and October) were chosen to represent each season, with specimens being curated and identified to the species level. Overall, our findings suggest that ant communities in peri-urban forests exhibit resilience to moderate anthropogenic disturbance, with differences in species diversity, community composition and abundances being not statistically significant between disturbed and undisturbed sites. These results are consistent with the intermediate disturbance hypothesis, highlighting that peri-urban forests can act as important reservoirs of biodiversity within urban landscapes. At the same time, the absence of non-native species highlights the need for long-term monitoring and management strategies that promote microhabitat diversity while mitigating the ecological risks associated with urbanization.

## Introduction

High species biodiversity offers advantages against the major changes in ecosystems caused by directional and stochastic phenomena (Chapin et al. 1998). In particular, arthropod diversity in an ecosystem plays an important role in many of its functions and is a source of multiple ecosystem services in the urban environment (Narango et al. 2018), such as food chains (Lewthwaite et al. 2024), decomposition processes, nutrient cycling, and biological control (Ossola et al. 2016). Human socio-economic activities in cities, which alter the biotic and abiotic characteristics of the environment, have a major impact on biological communities, particularly these of arthropods (Lewthwaite et al. 2024), which often undergo radical changes in species composition, abundance and richness (Shochat et al. 2006).

The total number and composition of species in a region are affected by various forms of human activity and the intensity of such activity (Chapin et al. 1998) and this effect can have complex consequences in local biodiversity (McKinney 2008). The negative effects of human development include the fragmentation of organisms’ habitats and the homogenization of the area in terms of vegetation, resulting in the loss of microhabitats that are essential for the preservation of biodiversity (Savard et al. 2000; Marzluff and Ewing 2001; McKinney 2008; Sattler et al. 2011). Human growth varies in intensity as we move from the center of urban cities to forested areas (Niemelä and Kotze 2009). Between these two extremes lie peri-urban areas, which often appear to have high biodiversity due to the creation of microhabitats, caused by the cultivation of ornamental plant species, irrigation, fertilizers and other human actions that alter the natural flow of resources in the ecosystem (Savard et al. 2000; McKinney 2008). This leads to the influx of non-native species, which may coexist for some time with the native ones and increase beta diversity (Niemelä 1999; McKinney 2002). This phenomenon is also supported by the intermediate disturbance hypothesis (Connell 1978), which states that in areas of moderate urbanization, species’ diversity is increased due to habitat heterogeneity (Blair 1996; Faeth et al. 2011).

Urban biodiversity in Greece appears to be greater than expected, as there are unrecorded microhabitats within cities that are home to many species (Kantsa et al. 2013). However, the stability of these ecosystems is reduced because their proximity to humans makes them vulnerable to fragmentation, degradation, and invasions. For example, Thessaloniki displays rich floral diversity in peri-urban forest areas, but the intense anthropogenic pressure is deteriorating their ecological quality (Petaloudi et al. 2022). The use of technologies such as remote sensing and citizen science can contribute to the conservation of urban biodiversity by helping to understand that land use and spatial planning play a more important role in urban areas than the availability of resources, thus demonstrating that green spaces function as refuges for life within the urban landscape (Ziliaskopoulos and Laspidou 2024; Tzortzakaki et al. 2019). Attica has undergone intense and continuous urbanization in recent decades, with urban coverage increasing from approximately 17.7% in 1945 to 68.5% in 1995 (Lasda et al. 2010), while between 1991 and 2016 there was a significant expansion of low-density urban areas (Gounaridis et al. 2018).

The aim of this research was to compare the composition and seasonal phenology of ant communities between undisturbed and disturbed areas of the peri-urban forest of Agia Paraskevi, highlighting the importance of understanding the effects of urbanization and its potential contribution to the conservation and management of peri-urban ecosystems. In this study, we evaluate ant community dynamics across a peri-urban disturbance gradient. Based on environmental filtering theory, we hypothesize that anthropogenic habitat modification acts as a strong ecological filter, replacing specialized soil-dwelling and leaf-litter species with disturbance-tolerant generalists due to micro-climatic desiccation and vegetation loss (Kraft et al. 2015; Ewers et al. 2015; Kotze et al. 2022). Based on ecological filtering and disturbance theory, we test three sequential predictions:

- P_1_ (Alpha Diversity): Species richness and diversity metrics (H’) will decline significantly with increasing human disturbance due to the loss of sensitive micro-habitats.
- P_2_ (Community Composition): Epigaeic ant community composition will differ significantly across disturbance regimes, marked by a structural shift from specialist-dominated to generalist-dominated assemblages.
- P_3_ (Beta Diversity Mechanics): Overall beta diversity across the gradient will be primarily driven by species turnover (replacement) rather than nestedness (loss/gain), as localized environmental filtering selects for distinct, disturbance-adapted species pools rather than simple species loss from natural sites.

While seasonal shifts drive baseline phenological patterns in invertebrate activity across Mediterranean-type ecosystems, localized human disturbance imposes persistent environmental filters on community assembly. Understanding how anthropogenic pressure interacts with underlying seasonal dynamics is essential for isolating reliable bioindicator metrics in rapidly changing peri-urban landscapes. In order to test our hypothesis, we used pitfall trapping data from June 2022 to May 2023, collected from areas of a peri-urban forest with detectable and non-detectable human activity (disturbed and undisturbed/natural areas) in the Agia Paraskevi region of Attica. Data on ants were examined for further analysis to explain how arthropod species communities and biodiversity patterns are formed in small Mediterranean peri-urban zones in Greece, which has been insufficiently studied (McIntyre 2000; Lewthwaite et al. 2024). While ants possess specialized functional traits that prevent direct extrapolation to broader arthropod taxa, their fine-scale sensitivity to soil and micro-habitat alteration makes them effective bioindicators of localized anthropogenic disturbance in peri-urban environments.

## Materials and methods

### Study area and period

Samples were collected over a period of one year (June 2022 to May 2023) from the area of Agia Paraskevi in Attica, Greece, specifically in the vicinity of Mount Hymettus (N38.004, E23.833). Situated in Attica, Mount Hymettus features a semi-arid Mediterranean climate with mean annual temperatures of 16.5–19.0 °C and around 400 mm of rainfall concentrated between October and April. Its elevation profile supports over 600 plant species, dominated by phrygana, maquis, and reforested *Pinus halepensis* stands. Sampling sites were selected along a defined peri-urban gradient to represent distinct intensity tiers of human disturbance. To ensure spatial independence among plots despite local proximity, each site was separated by major physical barriers (e.g., paved transport corridors, altered drainage structures, or distinct vegetative canopy shifts). Furthermore, micro-topographical features and substrate composition were documented for each plot to confirm habitat heterogeneity and preclude spatial autocorrelation among pitfall trap lines. In highly fragmented peri-urban environments, micro-habitat heterogeneity is frequently defined by sharp physical boundaries rather than linear distance. These structural features act as sharp micro-climatic barriers for epigaeic arthropods, maintaining distinct localized disturbance regimes and preventing direct ecological mixing between adjacent trap lines.

**Fig. 1:**
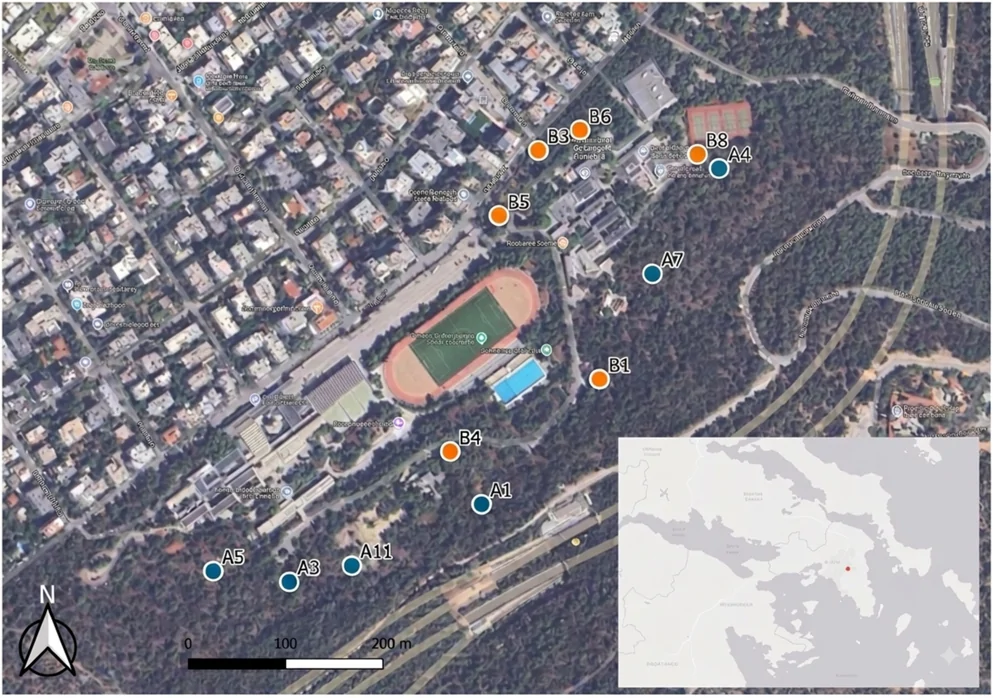
Spatial distribution of pitfall traps in the study area of Agia Paraskevi, Attica, Greece. Orange markers represent pitfall traps in disturbed sites and blue markers represent pitfall traps placed in undisturbed (natural) areas. The map uses the WGS 84 (EPSG:4326) Coordinate System. The inlay map (bottom right) indicates (as a red dot) the study area in relation to Attica. The figure was created using QGIS 3.36 (QGIS DT 2024). Map data © Google (n.d.), Maxar Technologies/Airbus

Samples were collected using pitfall traps made by using colourless cups of about 130 mm long and 76 mm outer diameter that were put inside a downpipe of 170 mm length and 80 mm outside diameter with 6 holes of 1 mm diameter drilled about 60 mm from the top, to prevent the cup from floating. A cover was also used above the pitfall traps to protect them from filling up with water from heavy rain and providing shelter from wild animals (e.g. foxes) with their rim flush with the ground level. Each trap was installed about 30 cm away from a characteristic woody plant (in most cases *Pinus halepensis* pine trees). Traps were placed at least 10 m away from each other. The pitfall traps were monthly changed (Fig. 1). For the purpose of examining seasonal variation, four representative months were selected: July (summer), October (autumn), January (winter) and April (spring), each corresponding to a season around the year. The months selected correspond to the mid-month of every season. Twelve (12) pitfall traps with propylene glycol were set, changed monthly and used in this analysis; six from areas at the center of human activity and six from natural areas (Fig. 1; Table 1). The selection of a subset of pitfall traps (from the 22 total pitfall traps initially placed) was necessary due to the time constraint of the first author sorting, curating and identifying the specimens collected within the time allowed for her final research project. In addition, the selected pitfalls were chosen so as to be spread out around the two areas; thus containing as many representative niches as possible. Data, such as the activities taking place on each site, the biological and non-biological materials found, vegetational cover, topographic and geospatial data, were recorded and separate labels were created for each sample (Table 1).

### Material examination

Bulk pitfall samples were fully sorted to account for all collected invertebrate material. Subsequent analyses focused exclusively on Formicidae, which comprised the most abundant terrestrial taxa across all traps and provided the sampling density required for robust spatio-temporal comparisons. Samples were classified into morphotypes, preserved in 95° ethanol and subsequently identified to the species-level based on the identification keys of Salata et al. (2019), Borowiec and Salata (2022, 2025), as well as expert consultation (Prof. Emeritus Lech Borowiec and Assoc. Prof. Sebastian Salata). Specimens are deposited at the Zoology Museum of the University of Athens (ZMUA).

### Statistical analysis

Ant biodiversity data from pitfalls deployed on July (summer), October (autumn), January (winter) and April (spring) were pooled as a presence/absence matrix (1 = present/0 = absent) and an abundance matrix (number of individuals per species per site) and were separately statistically analyzed in R Studio (version 2025.09.0) (Posit team 2025) using the *vegan* (Oksanen et al. 2024), *indicspecies* (De Cáceres and Legendre 2009), *ggplot2* (Wickham 2016), *ggrepel* (Slowikowski 2024), *dplyr* (Wickham et al. 2026), *ggpubr* (Kassambara 2025), and *betapart* (Baselga et al. 2025) packages. Coding was enhanced with the help of AI tools (Microsoft Copilot 2026) (MS 2026). Statistical analyses were executed to sequentially evaluate predictions P_1_ through P_3_:

Alpha Diversity & Richness (P_1_): To test whether species richness and Shannon diversity (H’) decreased along the disturbance gradient (P_1_), generalized linear mixed-effects models (GLMMs) were fitted with disturbance level and season as fixed factors and plot ID as a random effect. We calculated species diversity, the Shannon and Simpson diversity indexes separately for the presence/absence and abundance matrices. Boxplots were generated using custom *ggplot2* functions to compare diversity metrics between disturbed and undisturbed habitats. Statistical comparisons were performed using non‑parametric tests where appropriate.

To examine whether species richness and abundances varied across disturbed and undisturbed sites over the season (July, October, January and April), we constructed a presence/absence matrix and number of individuals matrix of species collected from each site during each period. We then pooled the number of species and abundances per site creating a database including: the site code (A1, A3, A4, A5, A7, A11, B1, B3, B4, B5, B6, B8), disturbance (disturbed/undisturbed), month, total species richness and species abundance. Using the *tidyverse* (Wickham et al. 2019), *lme4* (Bates et al. 2015), and *lmerTest* (Kuznetsova et al. 2017) packages, we performed a Mann-Whitney U test and a simplistic linear-mixed effect model with month and disturbance as fixed effects and site as a random effect. Community Composition (P_2_): Patterns in community composition were visualized using non‑metric multidimensional scaling (NMDS) (Kruskal 1964) based on Bray–Curtis dissimilarity for both the presence/absence matrix and the abundance matrix separately, using the function *metaMDS()* (*vegan* package), with two dimensions (k = 2) and 999 random starts. Site scores and species scores were extracted for visualization, and convex hulls were drawn around habitat groups to illustrate multivariate separation between disturbed and undisturbed sites. Differences in community composition were tested using permutational multivariate analysis of variance (PERMANOVA) with 999 permutations, to evaluate whether disturbance affected community structure differently depending on the data type. Effect sizes were expressed as R², representing the proportion of variation explained by habitat category.

To identify species associated with each habitat type, we used Indicator Value Analysis (IndVal and IndVal.g) (De Cáceres and Legendre 2009) using 999 permutations.

Beta Diversity Partitioning (P_3_): To determine whether community dissimilarity between natural and disturbed habitats stems from species replacement or nestedness (P_3_), total pairwise Sørensen dissimilarity (β_sor_) was partitioned into its spatial turnover (β_sim_) and nestedness-resultant (β_nes_) components using the *betapart* package following Baselga (2010). This distinguished whether disturbed communities represent impoverished subsets of natural sites or distinct assemblages driven by species replacement. Group differences in β_sim_, β_sne_, and β_sor_ were tested using permutation tests with 999 permutations to evaluate whether disturbed and undisturbed sites differed in their underlying beta‑diversity structure.

For P_2_ and P_3_, community dissimilarity was evaluated using two complementary approaches: Sørensen dissimilarity (binary Bray–Curtis) for presence/absence data to assess compositional turnover independent of group size, and abundance-weighted Bray–Curtis dissimilarity to capture shifts in relative species dominance across sampling sites.

All datasets, including presence/absence and abundance data per site, month and associated code can be found in Figshare (https://doi.org/10.6084/m9.figshare.31384306*).*

**Table 1:** Table of sampled localities including locality codes, decimal latitude and longitude, locality category as binary (disturbed/undisturbed), dominant woody plant and habitat notes e.g. disturbances

Locality code	Latitude	Longitude	Disturbed/Undisturbed	Habitat notes - Human disturbances
A1	38.001983	23.831867	Undisturbed	Detritus - Invasive plants - Dry grasses
A3	38.001267	23.829617	Undisturbed	Detritus
A4	38.005067	23.83465	Undisturbed	Detritus
A5	38.001367	23.828733	Undisturbed	Detritus
A7	38.0041	23.833867	Undisturbed	Detritus - Rocky environment - Mosses - Lichens
A11	38.001417	23.83035	Undisturbed	Moss - Mainly *Cistus*
B1	38.003133	23.83325	Disturbed	Gardening activities - Drilling - Student activities - Old garden - Food - Trash - Animals
B3	38.005233	23.832533	Disturbed	Fragmented area - Next to the Road - Student activities - Trash - Gardening activities
B4	38.002467	23.8315	Disturbed	Gardening activities - Next to the road inside campus - Trash - Animals
B5	38.004633	23.832067	Disturbed	Fragmented area - Student activities - Trash - Gardening activities
B6	38.005417	23.833017	Disturbed	Fragmented area - Student activities - Trash - Gardening activities
B8	38.0052	23.834383	Disturbed	Small parking - Student activities - Gardening activities - Trash

## Results

A total of 24 species were identified, with 19 and 21 taxa being recorded from undisturbed and disturbed sites, respectively (Table 2). Amongst them, no non-native species were detected while only one endemic species i.e. *Camponotus laconicus* Emery, 1920, was found (Table 2). Based on the latest available data all species are common and widespread throughout Greece (Salata and Borowiec 2017, 2019; Borowiec and Salata 2022, 2025).

**Table 2:** Ant biodiversity (presence/absence and abundances) collected from undisturbed and disturbed sites through pitfall sampling in Agia Paraskevi (Attica)

Site type/code	Presence/Absence		Abundance	
Species	Undisturbed	Disturbed	Undisturbed	Disturbed
***Aphaenogaster balcanica***	1	1	175	137
***Aphaenogaster epirotes***	1	1	7	15
***Aphaenogaster subterranea***	0	1	0	7
***Aphaenogaster subterraneoides***	1	0	2	0
***Camponotus ionius***	1	1	210	58
***Camponotus kiesenwetteri***	1	1	2	3
***Camponotus laconicus***	1	1	8	198
***Cataglyphis hellenica***	1	1	1	30
***Crematogaster ionia***	1	1	135	200
***Crematogaster sordidula***	1	0	1	0
***Lasius brunneus***	0	1	0	2
***Lepisiota frauenfeldi***	1	1	11	10
***Messor hellenius***	1	0	2	0
***Messor wasmanii***	1	1	82	11
***Pheidole pallidula***	1	1	33	117
***Plagiolepis pallescens***	1	1	5	4
***Plagiolepis perperamus***	0	1	0	8
***Plagiolepis pygmaea***	1	1	7	21
***Solenopsis juliae***	1	1	4	21
***Tapinoma glabrella***	0	1	0	3
***Temnothorax flavicornis***	1	1	2	4
***Temnothorax recedens***	1	1	31	1
***Temnothorax rogeri***	0	1	0	1
***Tetramorium kephalosi***	1	1	16	16

**Fig. 2:**
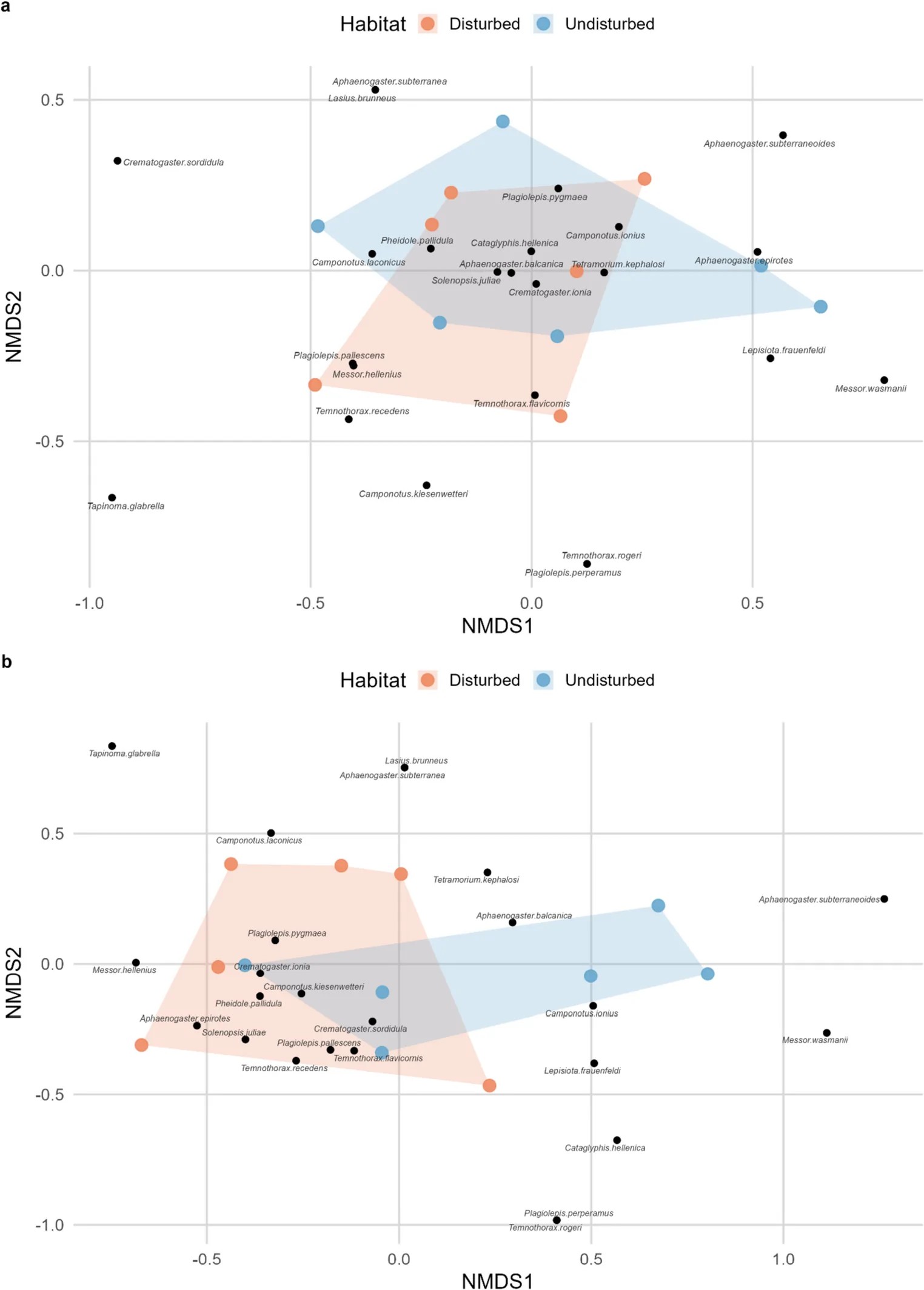
Non‑metric multidimensional scaling (NMDS) based on Bray–Curtis dissimilarity for the presence/absence matrix (Stress point = 0.169) (**a**) and the abundance matrix (Stress point = 0.122) (**b**)

The NMDS analysis showed that there is significant overlap between ant communities in disturbed and undisturbed areas (Fig. 2). Stress values of 0.169 (Fig. 2a) and 0.122 (Fig. 2b) were acceptable for further interpretation, although the PERMANOVA showed no significant partitioning of communities between disturbed and undisturbed sites for presence/absence (PERMANOVA, *p* = 0.684, R^2^ = 0.06) and abundance data (PERMANOVA, *p* = 0.073, R^2^ = 0.19). When using abundance data, disturbance explained approximately 20% of observed differences in community composition, with the p-value being statistically insignificant but close to the 0.05 threshold.

The Indicator Value Analysis did not identify any species associated with each habitat type, while beta diversity partitioning, applicable only for the presence/absence matrix (Baselga 2010), yielded an overall p-value for β_SIM_, β_SNE_ and β_SOR_ equal to 0.761, 0.779, and 0.728, respectively. Thus, indicating that disturbance did not significantly affect species turnover, nestedness and overall community dissimilarity between habitats.

**Fig. 3:**
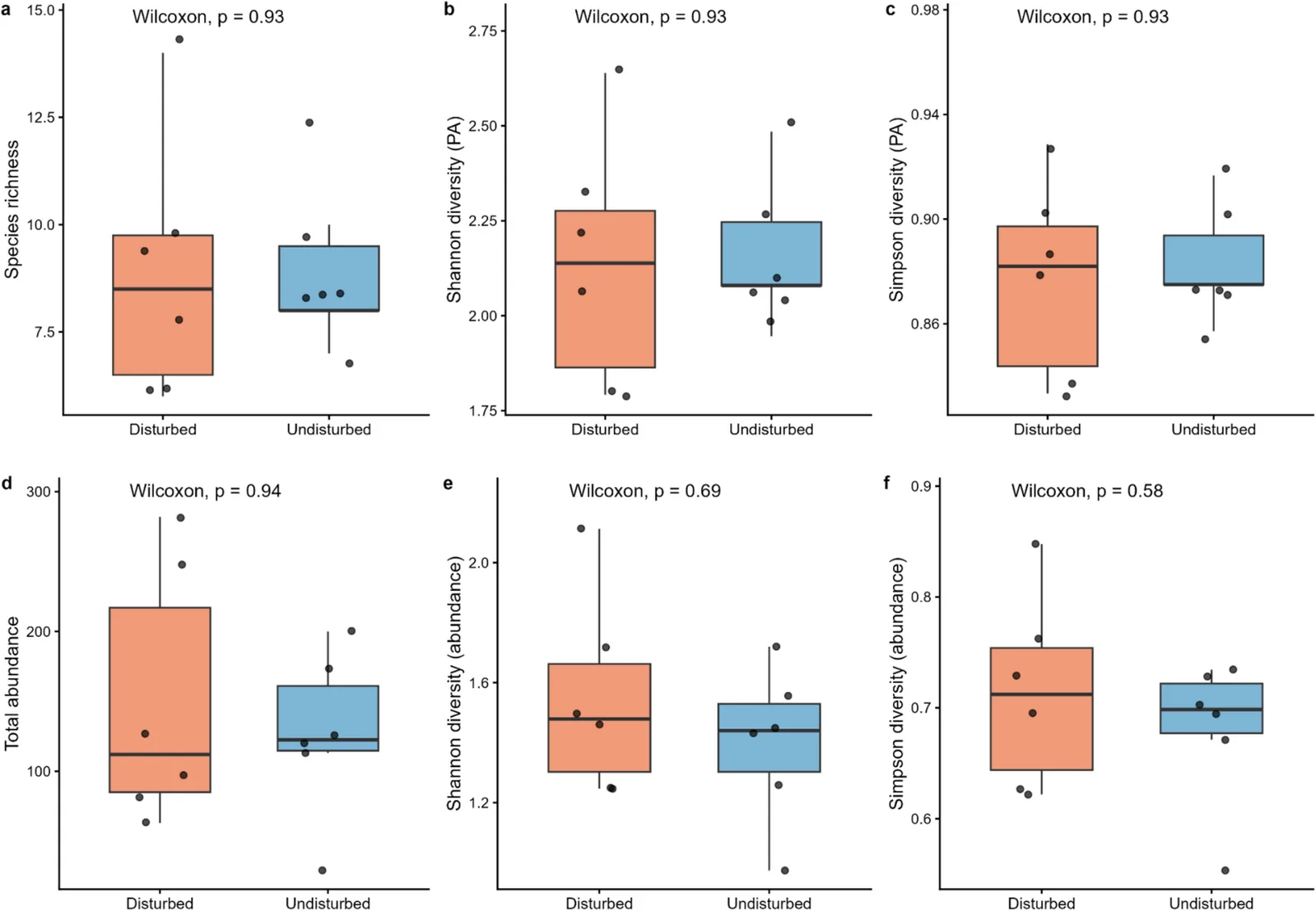
Boxplots depicting species richness (**a**), the Shannon (**b**) and Simpson diversity (**c**) indices when comparing the presence/absence matrix for disturbed and undisturbed habitats as well as total abundance (**d**), the Shannon (**e**) and Simpson diversity (**f**) indices when comparing the abundance matrix for disturbed and undisturbed habitats. The p-value using the Wilcoxon test is shown above each graph

No significant differences in species richness, the Shannon and Simpson diversity indices were found (Wilcoxon test) when comparing presence/absence matrices (Fig. 3a, b, c) and total abundances (Fig. 3d, e, f).

**Fig. 4:**
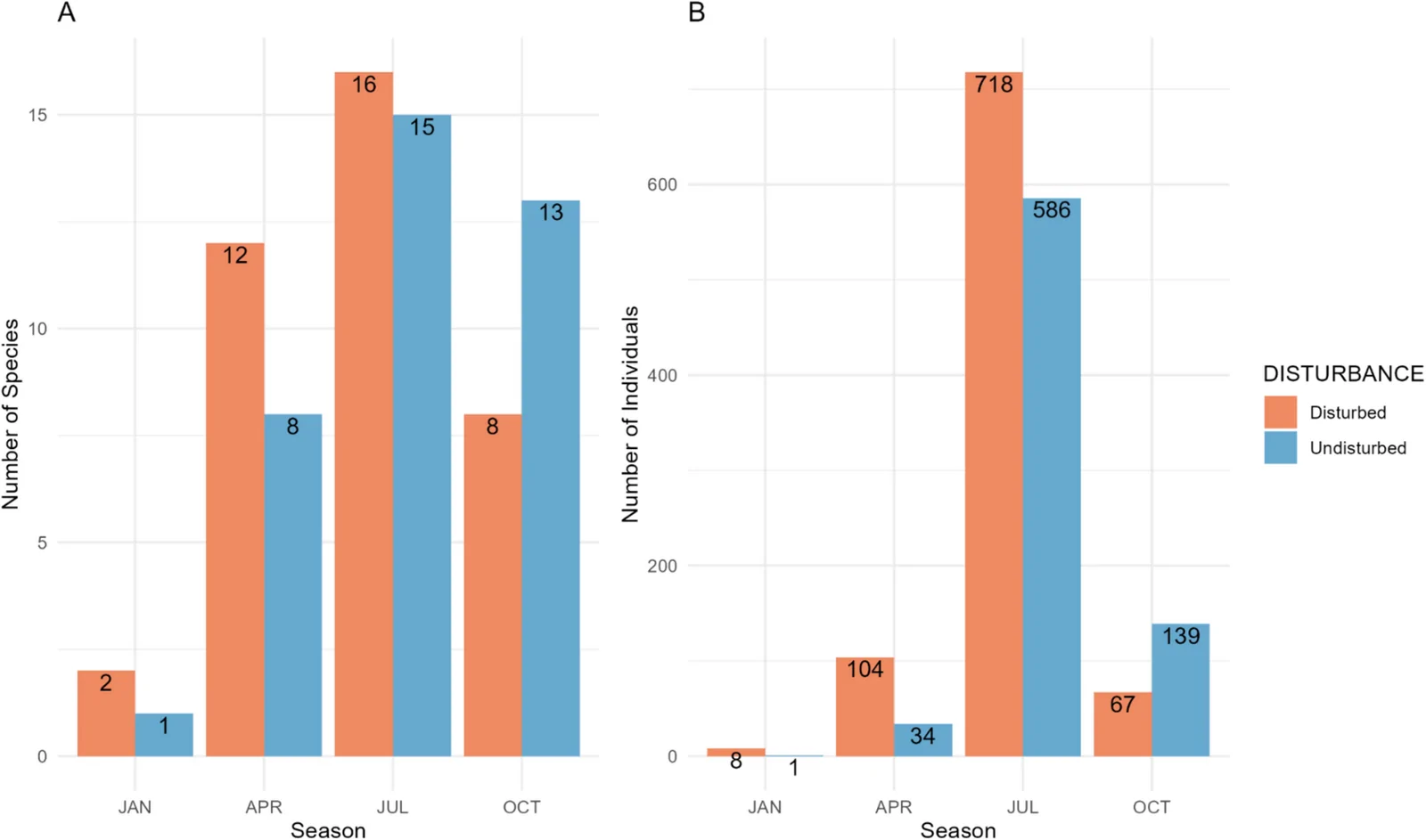
Number of species (**A**) and individuals (**B**) collected during each season in disturbed and undisturbed habitats

Although the number of species and individuals collected in disturbed and undisturbed sites varied between seasons (Fig. 4), the Mann-Whitney U test for both presence/absence (W = 16, *p* = 0.808) and abundance data (W = 17, *p* = 0.9362), as well as the linear mixed-effects model [effect of month (*p* < 0.001), disturbance (*p* = 0.5659) and month x disturbance interactions (*p* = 0.370) for presence/absence data and effect of month (*p* < 0.000001), disturbance (*p* = 0.62) and month x disturbance interactions (*p* = 0.71) for abundance data], showed no significant difference between the overall and seasonal species richness between disturbed and undisturbed sites.

## Discussion

This study examined ant diversity and community structure in a Mediterranean peri-urban forest, aiming to assess the impact of moderate human disturbance. Our findings indicate that, while differences were observed in the relative abundance of certain taxa, diversity indices and analyses did not reveal statistically significant differences between natural and disturbed areas. These results suggest that peri-urban habitats can sustain considerable ant diversity, even under moderate anthropogenic influence, and that moderate human disturbance in peri-urban areas may not necessarily erode community composition. In this case, ant assemblages in peri-urban forests appear to exhibit a somewhat significant degree of resilience as also witnessed by the lack of non-native species.

Patterns in community composition visualized using NMDS did not reveal a clear separation of samples by habitat type, with natural and disturbed areas overlapping. Partitioning of beta diversity revealed that differences in ant community composition between natural and man-made habitats were not driven by species turnover nor nestedness. This pattern suggests that ant communities are not strongly segregated along a natural-disturbed gradient but are instead shaped by a combination of seasonal variation and local heterogeneity. The occurrence of overlapping species between sites suggests that many taxa are flexible enough to benefit from both natural and anthropogenic environments. Similar findings have been reported in other urban ecology studies, where landscape mosaics and anthropogenic microhabitats contribute to mixed community structures and maintain elemental diversity in urban and peri-urban systems (McKinney 2008; Aronson et al. 2016; Socolar et al. 2016).

According to the intermediate disturbance hypothesis (Connell 1978), low to moderate levels of disturbance can promote species diversity by increasing habitat heterogeneity. In our case, activities in this peri-urban environment such as irrigation, planting and fertilization alter resource flows and create microhabitats that may temporarily favor native species. This process may locally increase beta diversity and help explain the observed mixing of communities in natural and disturbed areas. Nevertheless, the degree of disturbance has been also shown to facilitate the invasion success of non-native species compared to undisturbed ecosystems (Elton 1958; Hobbs and Huenneke 1992). While we found no non-native species of ants in the area, increased human activities may lead to the introduction, establishment and spread of non-native ants which could initially coexist with native species, but eventually displace them or alter ecosystem processes. Thus, the positive effects of moderate disturbance on diversity may be accompanied by long-term ecological risks, highlighting the need for continuous monitoring. Biological invasions of ants in Attica have not been adequately explored, with only four out of the 16 non-native ant species found in Greece, being reported for the Attica region (Borowiec et al. 2023; Demetriou et al. 2023). Amongst them, the invasive alien species *Linepithema humile* (Mayr, 1868), also known as the Argentine ant, which has been expanding its distribution in metropolitan Athens (Salata et al. 2019; Demetriou and Georgiadis unpubl. data). Further spread of the species towards peri-urban areas, facilitated by human-induced landscape modifications may well alter the observed biodiversity patterns and drastically reduce ant biodiversity in both disturbed and more natural areas (Cammell et al. 1996; Carpintero 2003; Carpintero et al. 2005; Centorame et al. 2017; Zina et al. 2020; López-Collar and Cabrero-Sañudo 2021).

When looking into seasonal species richness and abundances we observed the highest numbers in July, followed by April, October and January (Fig. 4). This may be attributed to climatological conditions allowing increases in colony abundances and wider foraging of individual ants from mid-spring to mid-autumn. A higher number of species and individual workers were collected from disturbed habitats in April and July. Although we acknowledge the caveats when dealing with abundance data from ants, this may also be attributed to anthropogenic activities e.g. irrigation and littering, providing food and humidity during the hot, dry season, which in turn help sustain larger numbers of species and individuals. These interventions augment soil moisture and resource heterogeneity, thereby generating additional microhabitats that can be utilized by ants. Similar findings have been reported in urban ecology studies, which highlight how anthropogenic habitat modifications can reinforce local resource availability and temporarily increase diversity (Narango et al. 2018; Lewthwaite et al. 2024).

Nevertheless, the opposite trend was observed in October, when the species richness and abundances of ants were larger in undisturbed habitats. This could be potentially explained by the early autumn rains freeing ants from having to forage in more anthropogenic habitats. However, when statistically checking for differences within disturbed and undisturbed sites we found no statistical significance in the role of disturbance in both the overall species diversity and species’ abundances. This re-inforces the results of the NMDS, with ant communities overlapping and foraging in both disturbed and undisturbed habitats without facing significant filtering of communities and taxa. These results provide additional evidence of the resilience of arthropod communities to moderate levels of anthropogenic pressure, consistent with observations in other urban and peri-urban ecosystems (Chapin et al. 1998; Ossola et al. 2016). While our study provides high-resolution data on local ant community responses across peri-urban disturbance gradients, we recognize the geographic constraints of single-region assessments. Expanding pitfall sampling networks across broader regional and biomic gradients will further validate the generalizability of these bioindicator metrics. Nevertheless, localized sampling schemes remain indispensable for identifying immediate, micro-scale ecological perturbations resulting from urban sprawl and human encroachment. A potential caveat in peri-urban bioindicator studies is the proximity of sampled plots across heterogeneous landscapes. However, our multivariate analyses confirm that community dissimilarity was driven by local disturbance severity rather than geographic spatial autocorrelation. Ground-dwelling ant assemblies respond strongly to micro-environmental conditions where even short physical distances across anthropogenic barriers (such as asphalt or compacted soil) can generate distinct species pools (Pećarević et al. 2010; Abdel-Dayem et al. 2021). While expanding pitfall trap networks across larger geographic scales will further refine these patterns, our targeted sampling successfully captures fine-scale community responses to localized urban disturbance.

The resilience of ant communities observed in this study suggests that peri-urban forests can operate as important reservoirs of biodiversity, even under moderate anthropogenic pressure. This observation reflects the complex role of anthropogenic influence, which does not always lead to direct biodiversity loss but can, under certain conditions, contribute to local habitat heterogeneity. Management strategies could focus on maintaining microhabitat heterogeneity while minimizing both direct (e.g. pollution/littering) and indirect habitat modifications (e.g. the introduction and spread of non-native species). Peri-urban areas can act as refuges within the urban grid, providing shelter for a wide range of arthropods, including ants. By preserving these areas, urban landscapes can maintain ecological connectivity and protect local biodiversity that might otherwise be lost due to ongoing urban expansion. Incorporating data from multiple seasons and years, combining multiple sampling methods and covering larger areas with varying levels of human interference, can help us better understand the realized dynamics of arthropod communities in peri-urban environments.

## Data Availability

No datasets were generated or analysed during the current study.
